# Biomimetic nerve guidance conduit containing engineered exosomes of adipose-derived stem cells promotes peripheral nerve regeneration

**DOI:** 10.1186/s13287-021-02528-x

**Published:** 2021-08-06

**Authors:** Zheng Yang, Yang Yang, Yichi Xu, Weiqian Jiang, Yan Shao, Jiahua Xing, Youbai Chen, Yan Han

**Affiliations:** 1 Department of Plastic Surgery, The First Medical Center, Chinese PLA General Hospital, Beijing, 100853 China; 2 Medical School of Chinese PLA, Chinese PLA General Hospital, Beijing, 100853 China; 3 Xi’an Daxing Hospital, Xi’an, 710016 Shaanxi China; 4 School of Medicine, Nankai University, Tianjin, 300071 China

**Keywords:** Peripheral nerve, Regeneration, Exosomes, Neurotrophin-3, Nerve guidance conduit

## Abstract

**Background:**

Efficient and stable delivery of neurotrophic factors (NTFs) is crucial to provide suitable microenvironment for peripheral nerve regeneration. Neurotrophin-3 (NT-3) is an important NTF during peripheral nerve regeneration which is scarce in the first few weeks of nerve defect. Exosomes are nanovesicles and have been served as promising candidate for biocarrier. In this work, NT-3 mRNA was encapsulated in adipose-derived stem cell (ADSC)-derived exosomes (Exo^NT-3^). These engineered exosomes were applied as NT-3 mRNA carrier and then were loaded in nerve guidance conduit (Exo^NT-3^-NGC) to bridge rat sciatic nerve defect.

**Method:**

NT-3 mRNA was encapsulated in exosomes by forcedly expression of NT-3 mRNA in the donor ADSCs. Exo^NT-3^ were co-cultured with SCs in vitro; after 24 h of culture, the efficiency of NT-3 mRNA delivery was evaluated by qPCR, western blotting and ELISA. Then, Exo^NT-3^ were loaded in alginate hydrogel to construct the nerve guidance conduits (Exo^NT-3^-NGC). Exo^NT-3^-NGC were implanted in vivo to reconstruct 10 mm rat sciatic nerve defect. The expression of NT-3 was measured 2 weeks after the implantation operation. The sciatic nerve functional index (SFI) was examined at 2 and 8 weeks after the operation. Moreover, the therapeutic effect of Exo^NT-3^-NGC was also evaluated by morphology assay, immunofluorescence staining of regenerated nerves, function evaluation of gastrocnemius muscles after 8 weeks of implantation.

**Results:**

The engineered exosomes could deliver NT-3 mRNA to the recipient cells efficiently and translated into functional protein. The constructed NGC could realize stable release of exosomes at least for 2 weeks. After NGC implantation in vivo, Exo^NT-3^-NGC group significantly promote nerve regeneration and improve the function recovery of gastrocnemius muscles compared with control exosomes (Exo^empty^-NGC) group.

**Conclusion:**

In this work, NGC was constructed to allow exosome-mediated NT-3 mRNA delivery. After Exo^NT-3^-NGC implantation in vivo, the level of NT-3 could restore which enhance the nerve regeneration. Our study provide a potential approach to improve nerve regeneration.

**Supplementary Information:**

The online version contains supplementary material available at 10.1186/s13287-021-02528-x.

## Background

Peripheral nerve injury is a common disease in clinical practice. Patients sustain neuropathic pain and deterioration of sensory, motor, autonomic function [1, 2]. Although peripheral nerve has potential for self-regeneration, long length of nerve defect, long duration before the treatment and several other factors limit its self-repair [3]. Also, spontaneous self-repair often lacks of adequate and directed axonal outgrowth and might be obstructed by scar formation. Direct neurorrhaphy could be applied only in the cases with short nerve gap without suture tension [4, 5]. Autologous nerve transplantation has been served as the “gold standard” for treatment of large nerve defect [6]. However, autologous nerve transplantation through microsurgical approaches is limited by the insufficient source of autologous nerves, considerable dysfunction of donor sites, mismatch between host nerves and grafts, and several complications [7]. With the increasing pursuit of alternative therapeutic strategies, various NGCs have been designed to provide mechanical, biological and biochemical supports. As NTFs are of great importance for Schwann cells (SCs) myelination, various NGCs are fabricated to deliver NTFs to provide suitable microarchitecture for axonal extension [8]. NT-3 is a critical autocrine NTFs of SCs, which supports SCs survival and differentiation, especially in the absence of axons. After peripheral nerve transection, the endogenous NT-3 dramatically decreases at 6 to 12 h after nerve injury and then remained at low level for at least 2 weeks due to persistent denervation induced by SCs dysfunction. Further experiments indicated that exogenous NT-3 supply promotes functional recovery of SCs and facilitate directional axon outgrowth, which shed light on the importance to the delivery of exogenous NT-3 [9, 10]. However, NT-3 delivery pose difficulties in maintaining its stability, retention duration, sustainable release, etc., therefore, to develop a novel NGC functionalized NT-3 delivery efficiently and effectively is worthy of consideration.

Exosomes are a kind of extracellular microvesicles with the diameter ranging from 30 to 200 nm [11], and exosomes are characterized by its easy manipulation, high biological penetration without immunogenicity, which renders exosomes as a potential strategy of therapeutic drug carrier [12, 13]. The ever-increasing evidence has shown that exosomes could encapsulate various bioactive molecules, such as RNAs, proteins, drugs, etc. [14, 15]; thereinto, mRNA could be encapsulated into isolated exosomes and then be endocytosed into the recipient cells to express targeting protein [16]. Unlike therapeutic protein delivery, the use of mRNA for the expression of target protein displays low immunogenicity, prolonged stability and potent translation.

Alginate is a kind of polysaccharide derived from algae, which possesses favorable biocompatibility and bears resemblance to the extracellular matrix (ECM) [17, 18]. Alginate scaffolds possess a highly porous structure with the pore sizes ranging between 100 and 150 μm in diameter which allows the transportation of cells within it [19]. Besides, ionotropic alginate hydrogel has been applied as a controlled release carrier for various bioactive cytokines in tissue regeneration, and it has been reported that small extracellular vesicles loaded within alginate hydrogel could prolong its retention [20–22].

Therefore, in this study, exosomes derived from ADSC were engineered to load with ample NT-3 mRNA. Encapsulated NT-3 mRNA could deliver to SCs stably and functionally. A biomimetic nerve guide conduit (Exo^NT-3^-NGC) containing alginate hydrogel loaded with Exo^NT-3^ as the intraluminal filler was constructed and expected to furnish contact guidance for axonal regeneration.

## Methods

### Cell culture

SC primary cultures were harvested as previously described with few modifications [23]; in brief, sciatic nerves of postnatal 1 day (P1) newborn Sprague Dawley rats were collected. After removing the epineurium, the nerves were cut into pieces. 0.03% type II collagenase and 0.25% trypsin (Sigma-Aldrich, USA) were used at 37 °C for 25 min to digest the nerves pieces and shaken evenly every 5 min. Then, the SCs were plated in 35 mm dishes cultured in Dulbecco’s modified Eagle’s medium (Thermo Fisher, USA) containing 15% fetal bovine serum and 1% penicillin–streptomycin solution (FCS; Thermo Fisher, USA) in a 37 °C with 5% CO_2_ and 92% humidity incubator.

Isolation of ADSCs was performed as previous described with few modifications [24]. Briefly, adipose tissues were collected from subcutaneous inguinal area of male Sprague Dawley rats at age of 12 weeks. After being cut into small pieces about 0.1 mm^3^, adipose tissues were digested with 0.1% type I collagenases (Sigma-Aldrich, USA) in Hank's Balanced Salt Solution containing calcium and magnesium (Hyclone, USA) at 37 °C for 1 h. Then, α-MEM (Hyclone, USA) containing 20% FBS (Gibco, USA) were neutralized digestive solutions. The collected solutions were combined with osmotic lysates (Beyotime, China) to remove red blood cells, afterwards, filtered by an 100 μm cell strainer. Finally, the cells were seeded on T-75 flasks in α-MEM containing 10% FBS and 1% penicillin/streptomycin (Gibco, USA) at 37 °C. The medium was changed every two days until the confluence of ADSCs were about 80–90%. The third to sixth passages of ADSCs were applied for experiments.

### Plasmid construction

The CDS of NT-3 cDNA was amplified by specific primers between Pac I (forward) and BstB I (reverse). The amplicon was digested and cloned into pWPI vector. The clones were verified by DNA sequencing and stored for following application. For PCR, primers were designed:

- forward: GGTTAATTAAGCCACCATGCTGGGCTTCCTGAAGA.
- reverse: GGTTCGAATCATGTTCTTCCGATTTTTC.

### Lentivirus packaging and virus infection

HEK293T cells were seeded in 6-well plates and cultured at the density of 60–80%. The cells were transfected with NT-3 plasmid together with the standard packaging plasmid (psPAX2) or enveloped plasmid (pMD2.G) at a 4:3:1 ratio by Lipofectamine 2000 (Thermo Fisher, USA) according to the manufacturer’s protocol. HEK293T cells were then incubated at 5% CO_2_, 37 °C for 48 h, and lentiviruses in supernatants were collected. ADSCs were seeded in 6-well plates at the density of 60–80%. The cells were infected with the lentivirus in medium containing 8 μg/ml polybrene (Sigma, USA). After 24-h infection, cells were replaced with fresh medium.

### Isolation and identification of exosomes

Exosomes were purified from ADSCs as previously described [25]. In brief, the medium of ADSCs culture medium was centrifuged under 500×*g* for 10 min to remove cells and cell fragments were removed at 12,000×*g* for 20 min. After filtered by a 0.22-μm filter, ADSCs-conditioned medium was harvested. Then, the supernatant fluid was centrifuged at 100,000×*g* for 30 min to obtain a concentrated liquor of ADSCs-derived exosomes. After centrifugation at 100,000×*g* for 120 min, the lower liquid layer was diluted by PBS and then centrifuged at 1000×*g* for 30 min. After washing three times in PBS, the resulting exosomes pellets were resuspended in PBS and frozen at − 80 °C for the experiments. The exosomes were imaged by transmission electron microscopy (JEM-200EX TEM, Tokyo, Japan). For absolute size distribution analysis, the exosomes were diluted to 500 ng/ml and then analyzed by Nanoplus (Gerbrunn, Germany). The proteins in exosomes were analyzed by western blotting using antibody against CD9 (ab92726, Abcam), GM130 (sc71165, Santa Cruz), TSG101(ab83, Abcam).

### Exosomes labeling and uptake assay

ADSCs-derived exosomes were labeled by 1,1′-dioctadecyl-3,3,3′,3′-tetramethylindocarbocyanine perchlorate (DiI; Beyotime, China) as previously described [26]. 100 μl exosomes were incubation 10 μM DiI at 37 °C for 15 min, and then washed with PBS at 100,000 g for 90 min at 4 °C. SCs were incubated with DiI-labeled exosomes (20 μg/ml) in a 35-mm confocal dish for 12 h. The cells were washed with PBS and fixed with 4% paraformaldehyde for 10 min and then incubated with S100 antibody (1:200, MA1-26621, Thermo Fisher, USA) at 37 °C for 1 h. After washing, the cells were incubated at room temperature for 1 h with Alexa Fluor-conjugated secondary antibodies (1:1000, Invitrogen, USA). Nuclei were dyed with 300 nM 4,6-diamidino-2-phenyl-indole (DAPI) for 5 min. The images were obtained using the confocal microscope.

### Analysis of exosomes release from exosome-alginate hydrogel and construction of NGC

Sodium alginate powder (Aladdin, China) was dissolved in PBS at 2% (wt/vol) at room temperature. 1% (wt/vol) calcium chloride solution was mixed and stirred at 1:4 volume ratio and then incubated at 37 °C for 10 min to form the exosome-alginate hydrogel. The isolated Exo^empty^ or Exo^NT-3^ (1 mg/ml) collected as described above was mixed evenly into the alginate solution at a volume ratio of 1:1. To quantify the indicated exosomes controlled release in alginate hydrogel, 100 μg exosomes loaded into 100 μl alginate hydrogel was immersed in 250 μl PBS solution (37 °C, 5% CO_2_ atmosphere), which was renewed for every two days. The PBS, containing the released exosomes from the hydrogel, was collected every two days to quantified cumulative released rate by using Bradford Protein Assay Kit. A 10-mm silicone tube with the inner diameter of 1.57 mm (Instech Laboratories, USA) was used as the external conduit of NGC. Exosome-alginate hydrogel was syringed into the external conduit to constructed the NGC. The constructed NGC with Exo^empty^- or Exo^NT-3^-alginate hydrogel was labeled as Exo^empty^-NGC and Exo^NT-3^-NGC, while the NGC without exosomes in alginate hydrogel was labeled as non-NGC.

### Animals and in vivo implantation

48 adult male Sprague Dawley rats at age of 12 weeks weighting 220 ± 20 g were randomly divided into 4 groups. Rats were anesthetized by 3% sodium pentobarbital solution (30 mg/kg, i.p.). The left lower limb was shaved and sterilized before the surgery. An incision about 2 cm was made parallel below the femur, the muscles were separated to expose the main branch of sciatic nerve. At the middle segment of the sciatic nerve trunk, a 10-mm defect was created, and the gap was bridged using the indicated NGC (labeled as non-NGC group, Exo^empty^-NGC group and Exo^NT-3^-NGC group) or by autologous nerve reversely sutured using 9-0 Polypropylene sutures (labeled as Autograft group). The muscles and incisions were closed by 3-0 nylon sutures. 2, 4 and 8 weeks after implantation, rats were killed randomly to detect the level of NT-3 mRNA of the proximal and distal segment in transected nerves. Motor function evaluation, electrophysiological test, morphological and immunofluorescence analysis were also performed.

### Polymerase chain reaction and western blotting

RNA was extracted using TRIzol reagent (Invitrogen, USA), reverse-transcription was used Transcriptor Reverse Transcriptase (Indianapolis, USA) under the guidance of manufacture instructions. Quantitative real-time PCR was performed by FastStart Essential DNA Green Master (Indianapolis, USA). Relative gene expression was normalized to GAPDH and quantified with the 2^ΔΔCt^ method for comparison.

Proteins were extracted from nerve tissues using 50 mM Tris–Cl, 150 mM NaCl, 100 μg/ml phenylmethylsulfonyl fluoride, protease and phosphatase inhibitor cocktail (#046931124001 and #4906837001, Roche, Switzerland), and 1% Triton X-100 on ice for 30 min. After centrifugation at 12,000×*g* at 4 °C for 15 min, the supernatant was collected and used for western blot analysis. Protein samples were separated by SDS-PAGE and transferred to a polyvinylidene fluoride membrane (Millipore, USA). Membranes were blocked with 6% nonfat milk in TBST for 1 h, and incubated with primary antibodies at 4 °C overnight. After washing membranes in TBST, membranes were incubated with secondary horseradish peroxidase-conjugated antibodies (1:5000) for 1 h at room temperature. Chemiluminescence regents (Millipore, USA) were used to visualize blots. Image Lab software (Bio-Rad Laboratories, USA) was used to detect the densities of immunoreactive bands.

### Hematoxylin and eosin analysis and immunofluorescence analysis

8 weeks after the implantation surgery, the animals were deeply anaesthetized by 3% sodium pentobarbital solution (30 mg/kg, i.p.). Regenerated nerves were collected for HE and immunofluorescence analysis. Paraffin-embedded tissue was cut into 5 μm slices, and hematoxylin and eosin staining was performed. Leica microscopy (JEM-1200 EX, Japan) was used to observe and photograph. Nerve tissues were also embedded in optimal cutting temperature (OCT) compound and immediately frozen in liquid nitrogen and then cut into 5 μm crysections. The samples were prefixed in 4% paraformaldehyde, then washed with PBS and incubated with S100 antibody (1:200, MA1-26621, Thermo Fisher, USA) and NF200 antibody (1:200, ab215903 Abcam, UK) at 37 °C for 1 h. After using PBS to wash the samples, secondary antibody was incubated for 1 h. Leica microscopy was used to visualize.

### Motor function evaluation after implantation

2 and 8 weeks after the implantation, walking track of animals was evaluated as previously described [27, 28]. In brief, rats were confined in a walkway 8.2 cm wide by 42 cm long with a dark shelter at the end. After two or three conditioning trials during which rats often stop to explore the corridor, rats walk then steadily to the darkened cage at the end of the corridor. The plantar surface of the rat hind feet has been dipped with red ink, in order to refine the prints for walking track analysis. The podogram length of normal and implantation was measured. SFI values calculated by Bain formula were applied (N: nonoperated group, E: experimental group, PL: podogram length, TS: distance between first and fifth toes or total spreading, IT: distance between intermediary toes).

The SFI value from − 100 to 0. − 100 implies thoroughly loss of motor function whereas neared 0 implies normal motor function.

### Electrophysiological test of regenerated nerves

The electrophysiological test was performed by Axon Digidata 1550 Digitizer, Molecular Devices as previously described [29–31]. In brief, 8 weeks after the implantation, the animals were deeply anaesthetized by 3% sodium pentobarbital solution (30 mg/kg, i.p.). One pair of stimulating electrodes was placed at the paraspinal site percutaneously where the sciatic nerve forms most proximally. The recording electrode needle was inserted into the lateral gastrocnemius muscle to recording the compound muscle action potentials (CMAPs) among consecutive stimulations. The recorded latency and amplitude of CMAP were analyzed.

### Statistical analysis

GraphPad Prism 6.0 software (San Diego, CA) was used to conduct statistical analysis. Quantitative data were showed as mean ± SEM. For the comparison of two groups was used Student *t* test. Comparing differences among three or more independent groups was tested by one-way ANOVA, and multiple comparisons were tested by Turkey’s post hoc test (*P* < 0.05).

## Results

### Isolation and characterization of engineered exosomes

**Fig. 1:**
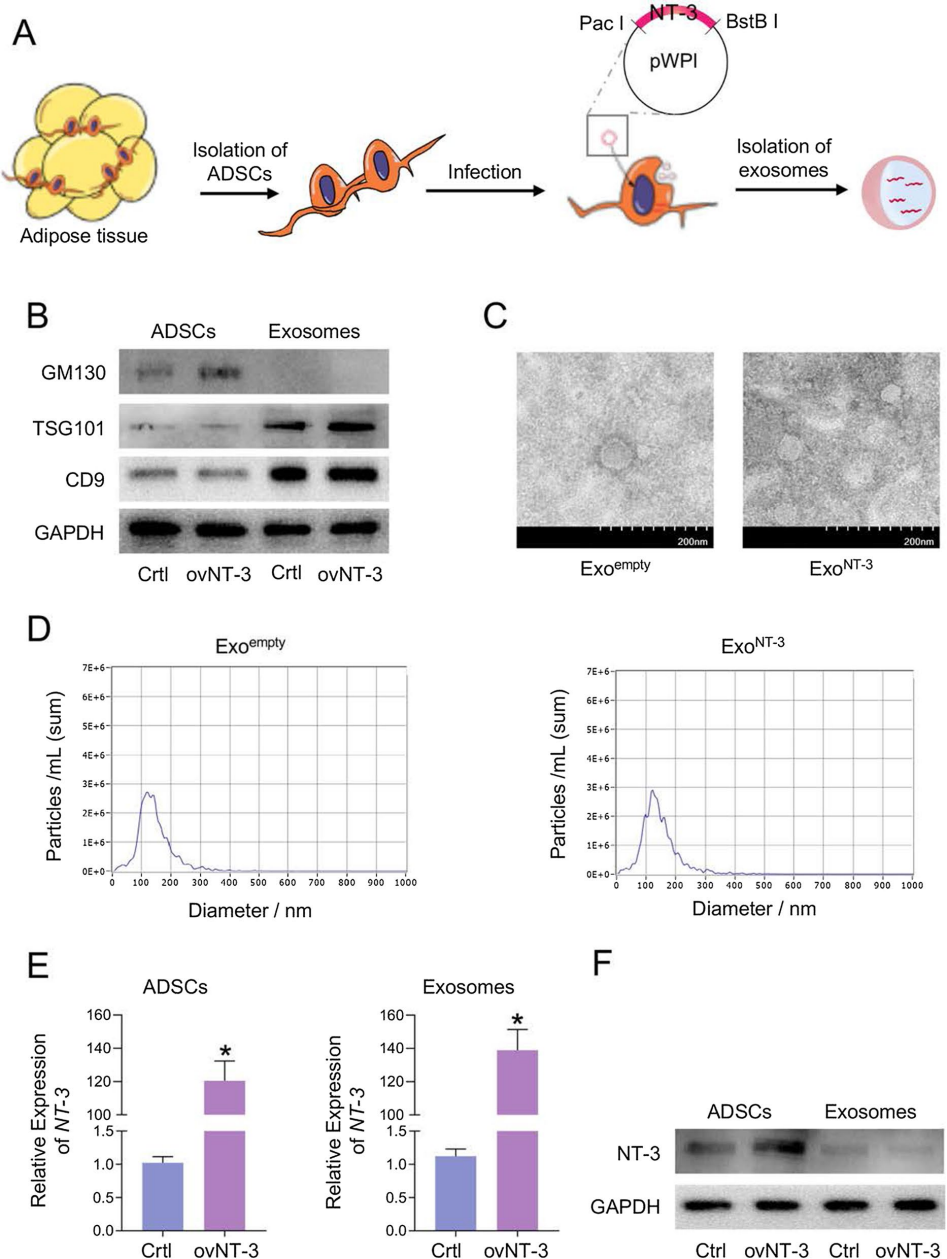
Construction and characterization of ExoNT-3. **a** Schematic illustration of NT-3 mRNA encapsulating into the ADSCs-derived exosomes. The sequence coding of NT-3 was package into the plasmid backbone which contained endonuclease sites as illustrated. ADSCs as the donor cell were compelled to express NT-3 through infection with NT-3 expressing virus. Therefore, NT-3 mRNA was enriched in the exosomes passively with the high level. **b** Representative western blotting presented the inclusive and exclusive marker of the isolated exosomes and the parental cells. ADSCs were infected with control or NT-3 vector. **c** Representative transmission electron microscopy images of the exosomes. **d** Size distribution of the exosomes. **e** Expression of NT-3 mRNA in ADSCs- and ADSCS-derived exosomes. GAPDH was tested as the internal control. **f** Representative western blotting of NT-3 protein expression in ADSCs and ADSCS-derived exosomes. ADSCs were infected with control or NT-3 vector. Data are presented as mean ± SEM in 3 different experiments, *P < 0.05 by *t* test

NT-3 mRNA-enriched exosomes were generated by constructing NT-3-expressing vector. Transcribed NT-3 mRNA could be passively loaded into the exosomes by means of the vector infection into the packaging ADSCs (Fig. 1a). Exosomal markers CD9 and TGS101 were highly expressed in both isolated exosomes of control (Ctrl) and NT-3 overexpression (ovNT-3) groups but low-expressed in ADSCs by Western blot. Golgi marker GM130, as a monitor of cellular contamination in exosome isolation, was only expressed in ADSCs while rarely detectable in Ctrl and ovNT-3 group of exosomes (Fig. 1b). Besides, nanoparticle tracking analysis and transmission electron microscopy showed similarity in size distribution and morphology between Exo^empty^ and Exo^NT-3^, ranging from 30 to 150 nm (Fig. 1c, d).

We further testified whether the NT-3 mRNA and protein were encapsulated into the exosomes successfully and efficiently. The results showed that NT-3 mRNA expression increased dramatically upon NT-3 overexpression in ADSCs, as well as in derived exosomes (Exo^NT-3^) (Fig. 1e). The level of NT-3 protein level remained low in either Exo^empty^ and Exo^NT-3^, while the increase expression of NT-3 protein was found in donor ADSCs (Fig. 1f). These results indicate that plenty NT-3 mRNA and mild NT-3 protein were encapsulated into the exosomes efficiently through its forced expression in the donor ADSCs.

### ***Exo***^***NT-3***^*** deliver functional NT-3 mRNA to SCs***

**Fig. 2:**
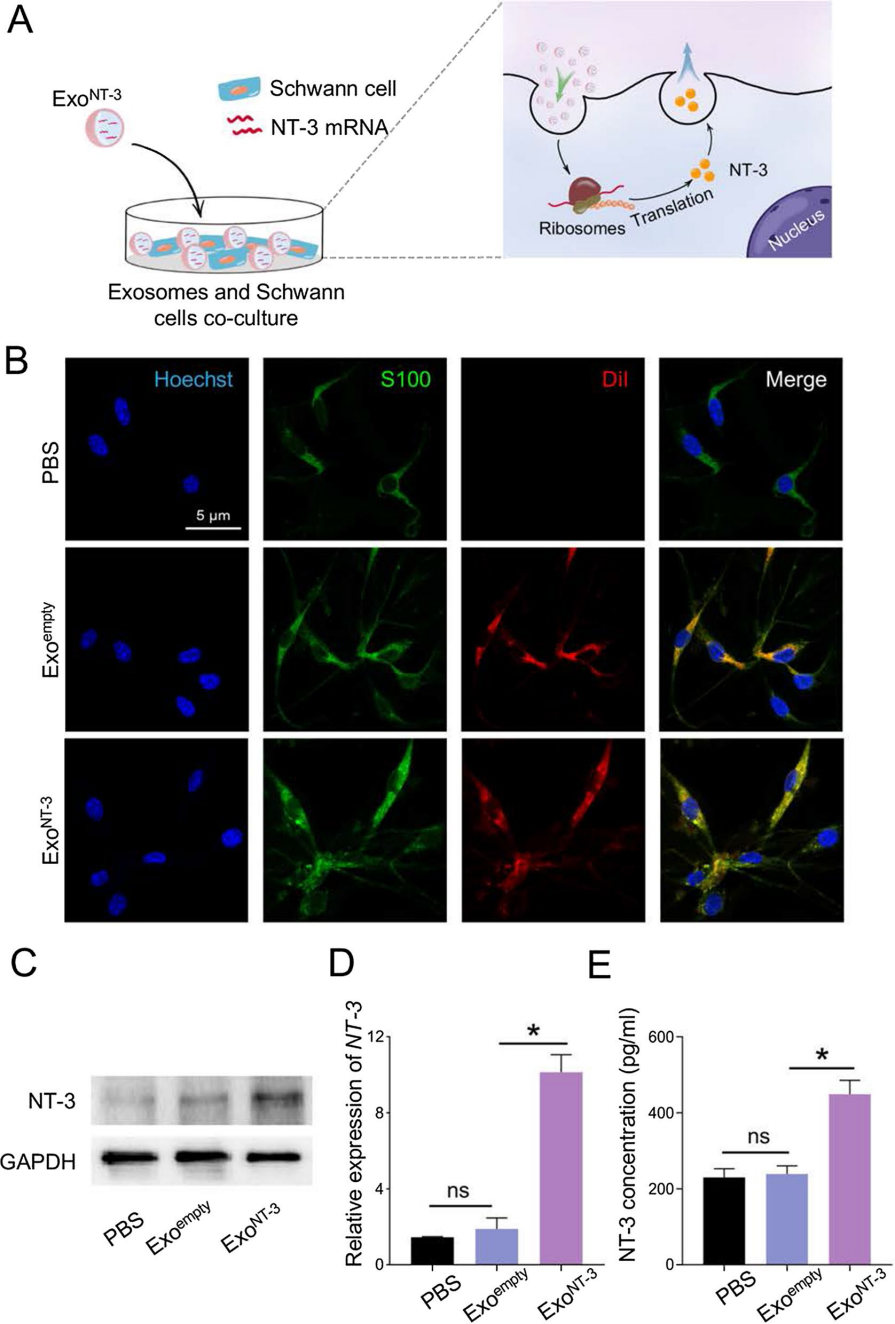
In vitro analysis of functional NT-3 mRNA delivery by ExoNT-3. **a** Schematic illustration of the process of NT-3 mRNA delivery by the exosomes into SCs, which then translated into functional protein. **b** Fluorescence confocal microscopy images exhibit the endocytosis of the exosomes by SCs. The intracellular distribution of the exosomes were tracked by using DiR label. S100 was used to label SCs and Hoechst was used to counterstain nuclei. PBS was cocultured with SCs as the negative comparison. **c** Representative western blotting of NT-3 protein expressing in SCs. **d** qPCR analysis of NT-3 mRNA expression level in SCs. **e** ELISA was performed to test the secretion of NT-3 in SCs. Data are presented as mean ± SEM in 3 different experiments, *P < 0.05 by one-way ANOVA

The Exo^NT-3^ was aimed to deliver NT-3 mRNA efficiently and then translated into functional protein in SCs so in order to support its growth. Therefore, Exo^empty^ and Exo^NT-3^ were stained with fluorescent dye DiI and then incubated with SCs (Fig. 2a). The distribution of exosomes was visualized by fluorescence microscopy. Exo^empty^ and Exo^NT-3^ were endocytosed by SCs significantly to similar extent (Fig. 2b). The remarkable increase in NT-3 expression level was showed in Exo^NT-3^ group accordingly, which had statistical significance (Fig. 2c). And there was also a mild increase in Exo^empty^ group with no statistical significance compared with control group. Similar pattern of NT-3 mRNA expression was also found in SCs (Fig. 2d). ELISA detection also found that SCs secreted an upregulated level of NT-3 in Exo^NT-3^ group compared with PBS and Exo^empty^ group (Fig. 2e).

The results above demonstrated that engineered Exo^NT-3^ could deliver NT-3 mRNA into targeted SCs, and could then be translated into NT-3 protein effectively, which upregulated NT-3 secretion of SCs.

### ***Preparation of Exo***^***NT-3***^***-NGC and in vivo implantation of NGCs***

**Fig. 3:**
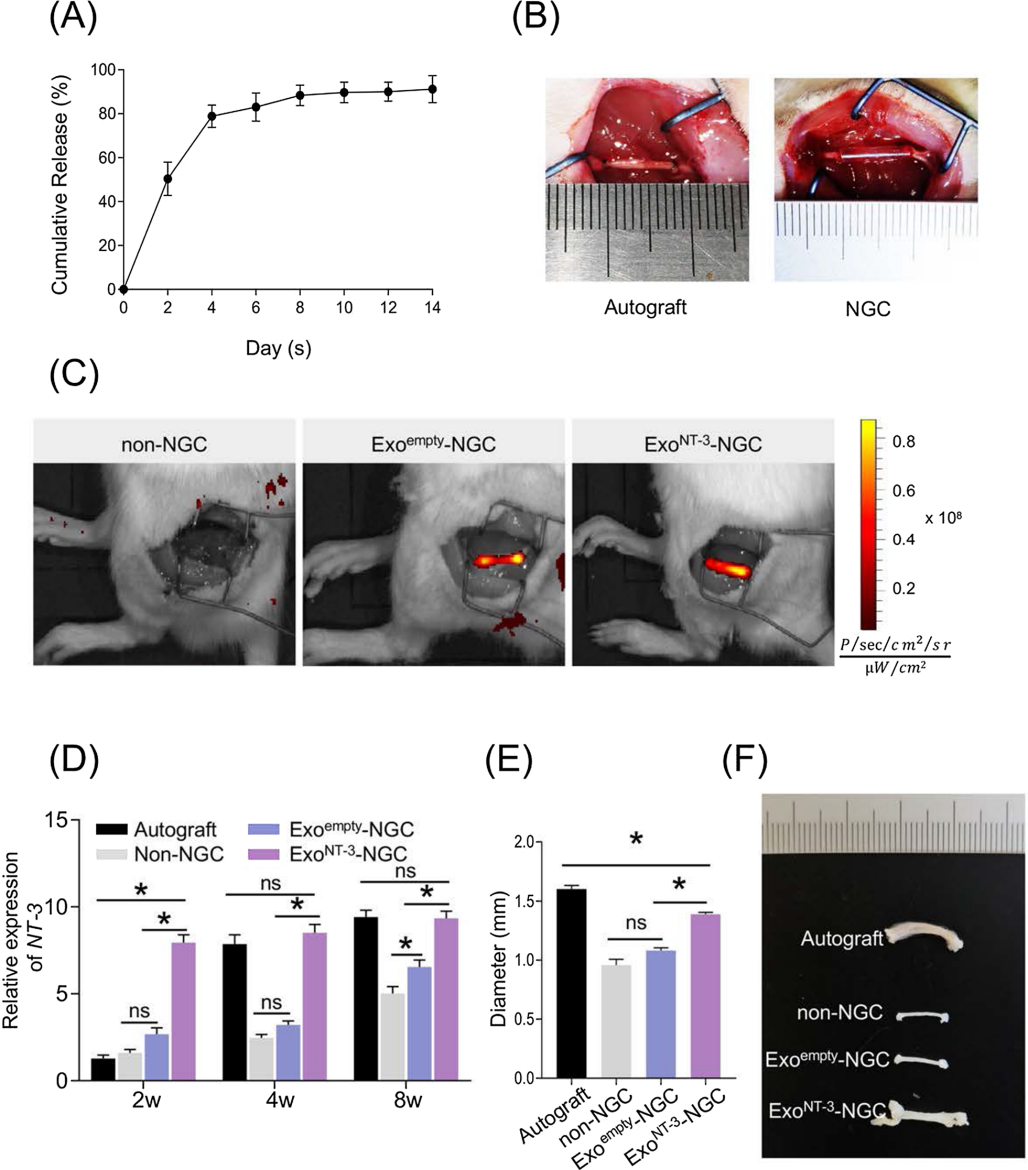
Construction and in vivo implantation of NGCs. **a** Exosomes release from alginate hydrogel. The cumulative release of exosomes was tested by the Bradford Protein Assay Kit. **b** NGCs were implanted in SD rat sciatic nerve defect model. Alginate hydrogel with Exo^empty^ or Exo^NT-3^ or without exosomes (1 mg/ml) was mixed (1:1 vol/vol) and syringed into 10 mm silicone conduits and then implanted in sciatic nerve defect in situ. Autogenous nerve inversed grafting served as the comparison. **c** Representative Caliper IVIS Lumina II images of rat. The exosomes were labeled DiR before NGCs construction. The rats were performed IVIS imaging after the implantation of NGCs. **d** qPCR analysis of NT-3 mRNA expression level in the distal segment of the transected nerves after 2, 4 and 8 weeks postoperation. **e** Statistical analysis of the diameter of regenerative nerve tissues after 8 weeks postoperation. **f** Gross observation of regenerative nerve tissues. Data are presented as mean ± SEM, *n* = 6 rats per group. *P < 0.05 by one-way ANOVA with post hoc Bonferroni correction

In the following experiments, alginate hydrogel contained Exo^NT-3^ was created to applied as intraluminal filler of the constructed NGC (Exo^NT-3^-NGC). The exosomes released in abundance in the first few days and then remained steadily in about 14 days (Fig. 3a). Alginate hydrogel combined with Exo^empty^ or Exo^NT-3^ were syringed into external conduits of the constructed NGCs. To evaluate the nerve regeneration guiding function in vivo, rats were divided into four groups, and 10-mm sciatic nerve defect model was established. NGCs were implanted into defect sites, and autograft implantation model was set as comparison (Fig. 3b). All experimental rats suffered implantation without adverse reaction. To visualize the exosomes in NGCs, DiR was used to label the exosomes. Both DiR-labeled Exo^empty^ and Exo^NT-3^ were gathered inside the NGCs at pre-established defect sites comparably (Fig. 3c).

2, 4 and 8 weeks after NGC implantation in vivo, the level of NT-3 mRNA of the proximal and distal segment in transected nerves were detected. According to the results, NT-3 mRNA of the distal segment remained the low level at 2 weeks after the operation in Autograft group, and dramatically increased after 4 weeks. Exo^NT-3^-NGC implantation led to NT-3 mRNA level increasing obviously, especially at 2 weeks after the implantation, and maintained at the high level even at 8 weeks which is comparable to Autograft group. Mild increase in NT-3 mRNA level was found in Exo^empty^-NGC group in first 4 weeks and increased to some extent at 8 weeks, while no obvious changes were observed before 4 weeks after the implantation in non-NGC group (Fig. 3d). And the proximal segment of NT-3 mRNA level showed similar pattern (Additional file 1: Fig. S1). 8 weeks after implantation, rats were killed, and the gross observation of regenerated sciatic nerve-like stumps were displayed (Fig. 3f). The diameter of the regenerated nerve tissues remained thin in Exo^empty^-NGC group, which had no significance with non-NGC group, while the regenerated nerve tissues in Exo^NT-3^-NGC group obviously get thicker than in non-NGC and Exo^empty^-NGC group, which was comparable to the Autograft group. (*P *< 0.05) (Fig. 3e).

### ***Exo***^***NT-3***^***-NGC promote nerve regeneration***

**Fig. 4:**
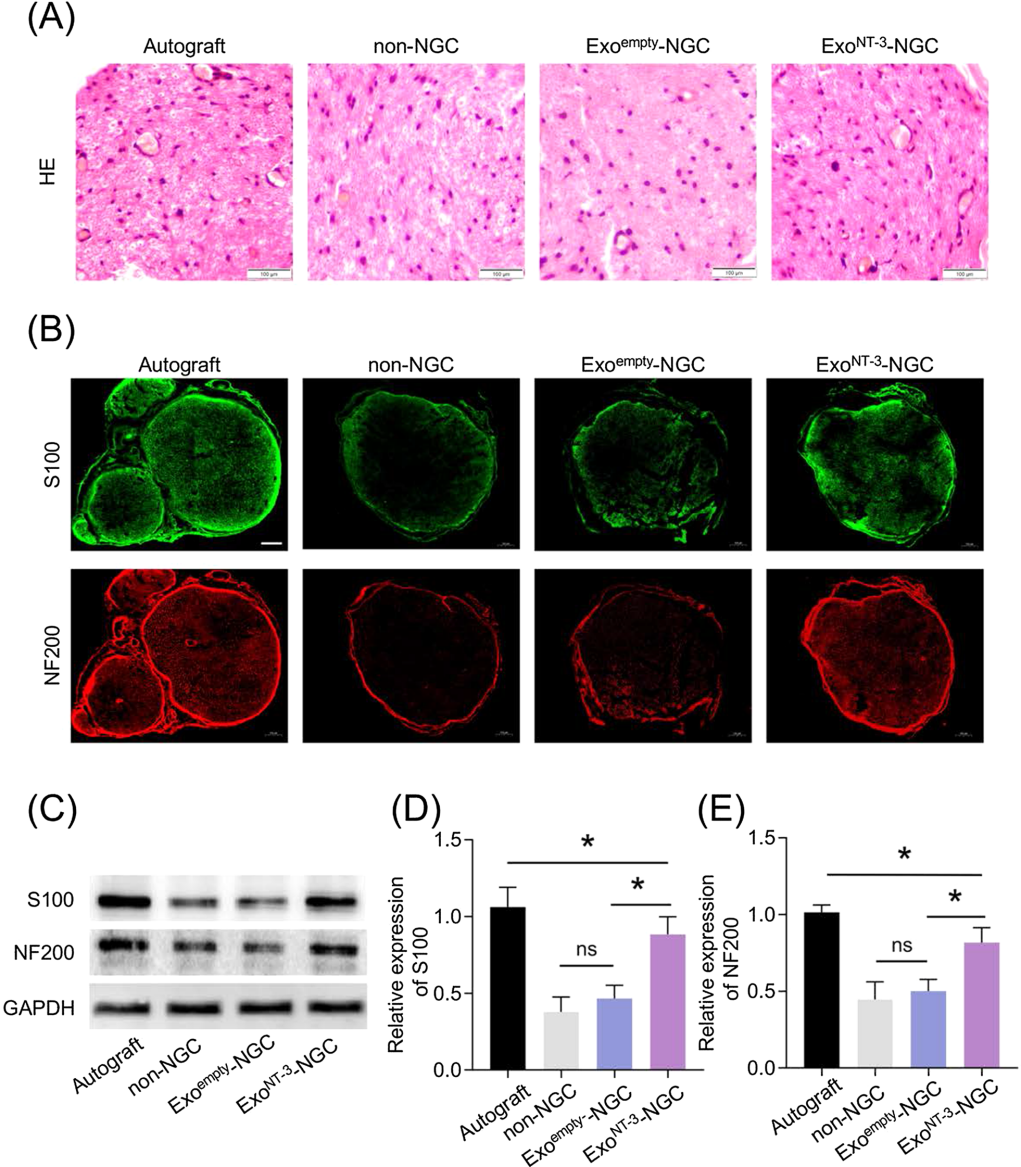
Evaluation of the regenerated nerves morphology and immunofluorescence analysis. **a** Cross section from the distal segments of regenerated nerves were stained by HE stain. The blue arrow indicates vessels. **b** Cross section from the distal segments of regenerated nerves were immunostained 8 weeks after the implantation. S100 indicated the regenerated SCs and NF200 indicated the regenerated axons. Scale bar = 100 μm. **c** Representative western blotting of S100 and NF200 protein expressing in regenerated nerve tissues. **d** Statistical analysis of S100 protein expression in regenerated nerve tissues. **e** Statistical analysis of NF200 protein expression in regenerated nerve tissues. Data are presented as mean ± SEM, *n* = 6 rats per group, *P < 0.05 by ANOVA with post hoc Bonferroni correction

The distal segments of regenerated tissues were observed by histological assessments. HE stain of fascicles showed numerous remyelinated nerve fibers adjacent to regenerated vessels and blood cells in Exo^NT-3^-NGC group, which is comparable to Autograft group, while in non-NGC and Exo^empty^-NGC group, SCs were sparse with few vessels and blood cells (Fig. 4a).

S100, as a marker of SC, is related to the secretion of NGF and the formation of myelin sheath. The expression of S100 is paralleled to the maturation of peripheral nerves [32]. NF200, as a marker of axon, has positive effects on axons mature which affects neuron size and the velocity of nerve conduction [33]. Immunofluorescence staining for regenerated nerves showed manifested positive expression of S100 and NF200 in Autograft group compared to obvious decrease in non-NGC and Exo^empty^-NGC group, while improved distributions of these two protein were showed in Exo^NT-3^-NGC group (Fig. 4b). Western blotting results showed the same expression pattern of S100 and NF200 (Fig. 4c–e).

### Regenerated nerves functional evaluation

**Fig. 5:**
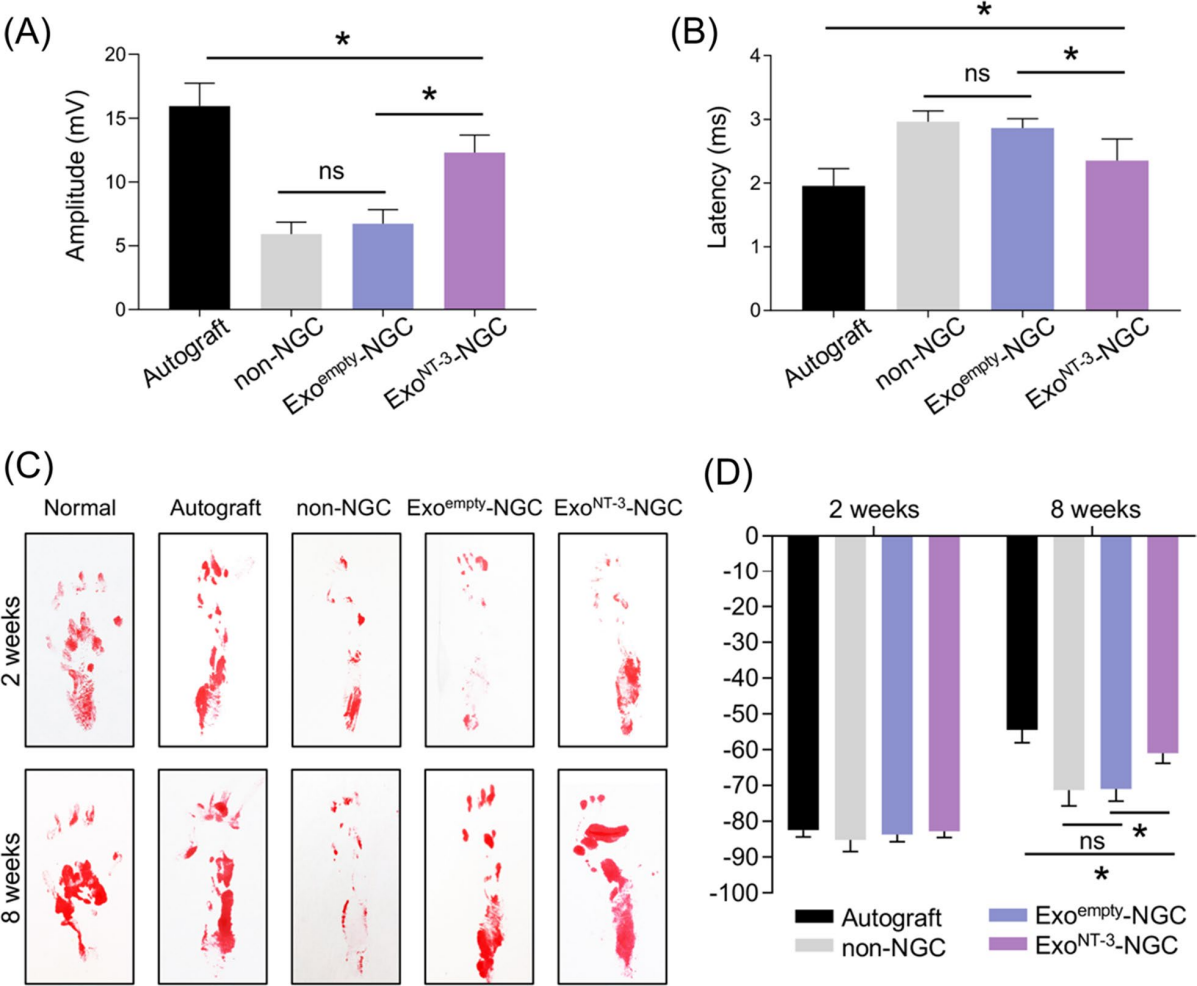
Functional evaluation of regenerated nerves. **a** Electrophysiological assessment of the sciatic nerve in each group was analyzed 8 weeks after implantation. Statistical analysis of amplitude and **b** latency was shown. **c** Motor function restoration was evaluated 2 and 8 weeks after implantation. Representative footprints were photographed. **d** Quantitative analysis of SFI values. Data are presented as mean ± SEM, *n* = 6 rats per group, *P < 0.05 by ANOVA with post hoc Bonferroni correction

The electrophysiological assay was used to evaluate the function recovery of regenerated nerves. The amplitude of compound muscle action potential (CMAP) could reflect the strength of nerve conductive ability [34]. The amplitude of CMAP indicates the number of regenerated axons which reach the targeted muscles. The myelination of regenerated nerves is closely related to the velocity of nerve conduction, which could be indicated by the latency of CMAP [35]. Quantification of CMAP manifested higher amplitude and shorter latency in Exo^NT-3^-NGC group compared to non-NGC and Exo^empty^-NGC group with distinctive statistical significance (Fig. 5a).

Sciatic nerve injury causes dysfunction of fibular nerve which leads to rats claudication, abnormality of plantar flexion, difficulty of ankle spread, and footprint length increase. Sciatic functional index (SFI) is the golden standard for the evaluation of motor function restoration of sciatic nerve after injury [36]. Compared to normal sides, the footprints of operated sides were shallow, and the toes were arched and curled together at the first 2 weeks after surgery in all groups with no statistical significance of SFI values. With the recovery last, 8 weeks after implantation, SFI values increased in all groups, while it increased more dramatically in Autograft and Exo^NT-3^-NGC group. The toes in Exo^NT-3^-NGC group were spread and the length of footprint shortened to a certain extent; as a result, SFI values reduced as a comparison to non-NGC and Exo^empty^-NGC group (*P* < 0.05) (Fig. 5c, d). These data demonstrate that the motor function of Gel-Exo^NT-3^ group rats evidently restored.

### Evaluation of gastrocnemius muscle

**Fig. 6:**
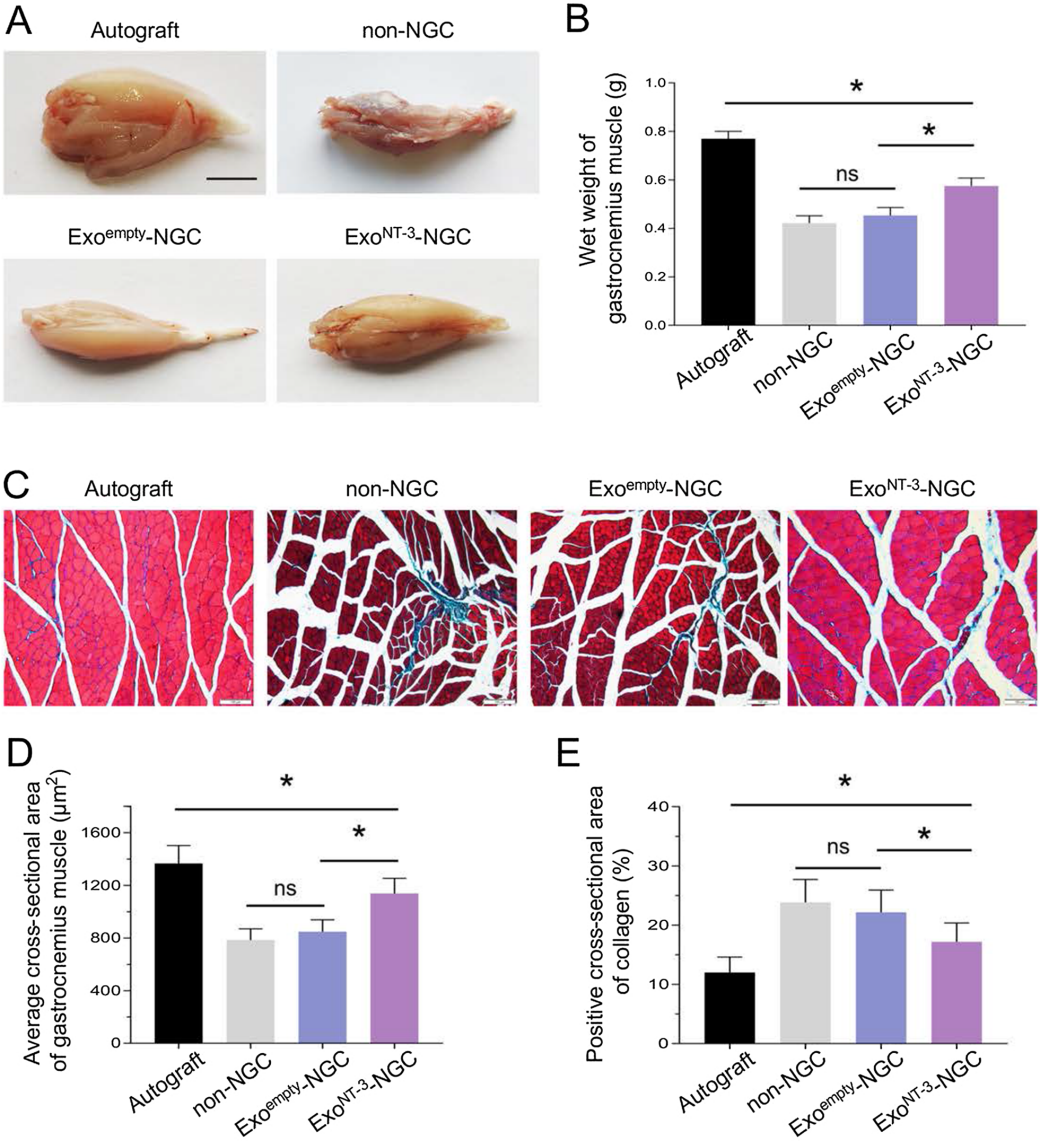
Evaluation of gastrocnemius muscle. **a** Gross observation of gastrocnemius muscle 8 weeks after implantation. Scar bar = 5 mm. **b** Statistical analysis of wet weight of gastrocnemius muscle. **c** Representative photomicrographs of gastrocnemius muscle. The cross sections of gastrocnemius muscle were stained by Masson’s trichrome. **d** Statistical analysis of myoatrophy was quantified by muscle fibers area and collagen fibers ratio. Data are presented as mean ± SEM, *n* = 6 rats per group, *P < 0.05 by ANOVA with post hoc Bonferroni correction

After sciatic nerve transection, gastrocnemius muscle, as the target muscle of sciatic nerve, becomes atrophy and dysfunctional attributed to long-term denervation. Denervated muscle shows weight decrease, and muscle fibers shrink combined with collagen fibers proliferation. After implantation, myoatrophy could be meliorated as nerve reinnervation [37]. 8 weeks after implantation, the wet weight of gastrocnemius muscle showed distinctive difference between Autograft group and other groups (*P* < 0.05). No significant difference between non-NGC and Exo^empty^-NGC group, while the wet weight apparently increased in Exo^NT-3^-NGC group (*P* < 0.05) (Fig. 6a, b). Masson’s trichrome staining was applied to visualize the histomorphology of gastrocnemius muscle (Fig. 6c). The sectional area of denervated muscle showed myofibers shrink accompanied by collagen fibers increase in non-NGC and Exo^empty^-NGC group, whereas in Exo^NT-3^-NGC group, myofiber loss was alleviated to some extent as well as collagen fiber decrease (Fig. 6d, e). These results demonstrated that Exo^NT-3^-NGC is more conducive to relieve the atrophy of gastrocnemius muscle during nerve regeneration process.

## Discussion

The growth and differentiation of SCs is crucial in the process of peripheral myelination and regeneration, and several NGFs are involved in survival, differentiation, maturation of SCs. NT-3 is mainly secreted by SCs and exerts autocrine regulation of SCs survival, differentiation and remyelination through its tyrosine kinase C (TrkC) receptor after peripheral nerve injury [38–40]. However, NT-3 decreases dramatically once nerve transection and sustains at low level for 2 weeks [41, 42]. Several studies have reported that exogenous NT-3 application, such as NT-3 injection [43], NT-3 carrier implantation [44], matrices loaded NT-3 [45], and mini-pump implantation [46]. Promote survival of denervated SCs, axonal regeneration and tissue repair. However, exogenous NT-3 application is prone to diffuse, and it is difficult to maintain a considerable concentration at injured site.

Recently, increasing studies have revealed that exosomes possess high physicochemical stability and biocompatibility with low toxicity and immunogenicity, which is of great potential as a biocarrier. Various cargos could be encapsulated into exosomes, such as nucleic acids and proteins, and then be transported to recipient cells to trigger intracellular changes, which sets the basis of exosome-based therapy [47]. The use of mRNA to expression of therapeutic proteins might have a wider range of application. mRNAs designed to expression target proteins display prolonged stability, low immunogenicity, and functional translation. Exosomes-based intracellular mRNA delivery allows the expression of any desired protein in recipient cells and tissues with preservation of post-translational modification. Besides, exosome-mediated mRNA delivery could overcome many limitations posed by naked mRNA delivery, such as passive diffusion by cell membrane, immediate extracellular degradation by ubiquitous RNases, limited endocytosis [48].

As NT-3 is an important autocrine factor of SCs which needs intracellular post-translational modification to realize its biological function, and the delivery of NT-3 mRNA could be more rational and efficient to recipient cells [49, 50]. In this study, ADSCs-derived exosomes were engineered to encapsulated with NT-3 mRNA, which could then be translated into bioactive NT-3 protein in SCs. Then, we created alginate hydrogel incorporated with exosomes as the intraluminal filler of the constructed NGC. The intraluminal filler could stably release the exosomes for at least 2 weeks, which is happened to remedy the low level of NT-3 after peripheral nerve injury. Besides, alginate as fillers also has a beneficial effect on SCs adhesion, proliferation and migration [51, 52]. Thus, the constructed NGC with intraluminal filler might not only provide mechanical support but also endogenous NGF supply.

## Conclusion

In this study, we engineered exosomes to deliver NT-3 mRNA to SCs to restore NT-3 level. This strategy could serve as an efficient way for endogenous supply of NT-3. The NGC with NT-3 encapsulated exosomes as the intraluminal filler could promote nerve regeneration in bridging the defect of rat sciatic nerve. We provide a novel strategy for the construction of NGC to furnish the desirable microenvironment for peripheral nerve regeneration.

## Supplementary Information

**Additional file 1: Fig. S1**. qPCR analysis of NT-3 mRNA expression level in the proximal segment of the transected nerves after 2, 4 and 8 weeks postoperation. Data are presented as mean ± SEM, *n* = 6 rats per group. *P < 0.05 by one-way ANOVA with post hoc Bonferroni correction.

## Data Availability

The datasets of current study are available from the corresponding author for reasonable request.

## Abbreviations

ADSCs: Adipose-derived stem cells
SCs: Schwann cells
NGC: Nerve guidance conduit
SFI: Sciatic Functional Index
NTFs: Neurotrophic factors
NT-3: Neurotrophin-3
