# Supplementary material for: Biomimetic nerve guidance conduit containing engineered exosomes of adipose-derived stem cells promotes peripheral nerve regeneration
Source: Stem Cell Res Ther. 2021 Aug 6;12:442. doi: 10.1186/s13287-021-02528-x (PMC8343914; doi:10.1186/s13287-021-02528-x)
Supplement: Supplementary file 1 — Additional file 1: Fig. S1. qPCR analysis of NT-3 mRNA expression level in the proximal segment of the transected nerves after 2, 4 and 8 weeks postoperation. Data are presented as mean ± SEM, n = 6 rats per group. *P < 0.05 by one-way ANOVA with post hoc Bonferroni correction. [file 13287_2021_2528_MOESM1_ESM.docx]

**Supplemental Files**

**Biomimetic nerve guidance conduit containing engineered exosomes of Adipose derived stem cells promote peripheral nerve regeneration**

Zheng Yang^1,2†^, Yang Yang^3†^, Yichi Xu^1^, Weiqian Jiang^1,2^, Yan Shao^4^, Jiahua Xing^4^, Youbai Chen^1*^,Yan Han ^1*^

* Correspondence: youbaichen@foxmail.com; [13720086335@163.com](mailto:13720086335@163.com)

^†^Equal contributors

^1^Department of Plastic Surgery, The First Medical Center, Chinese PLA General Hospital, Beijing 100853, China.

^2^Medical School of Chinese PLA, Chinese PLA General Hospital, Beijing 100853, China.

^3^PLA 82nd Group Army Hospital, Hebei Baoding 071000, China.

^4^School of Medicine, Nankai University, Tianjin 300071, China.


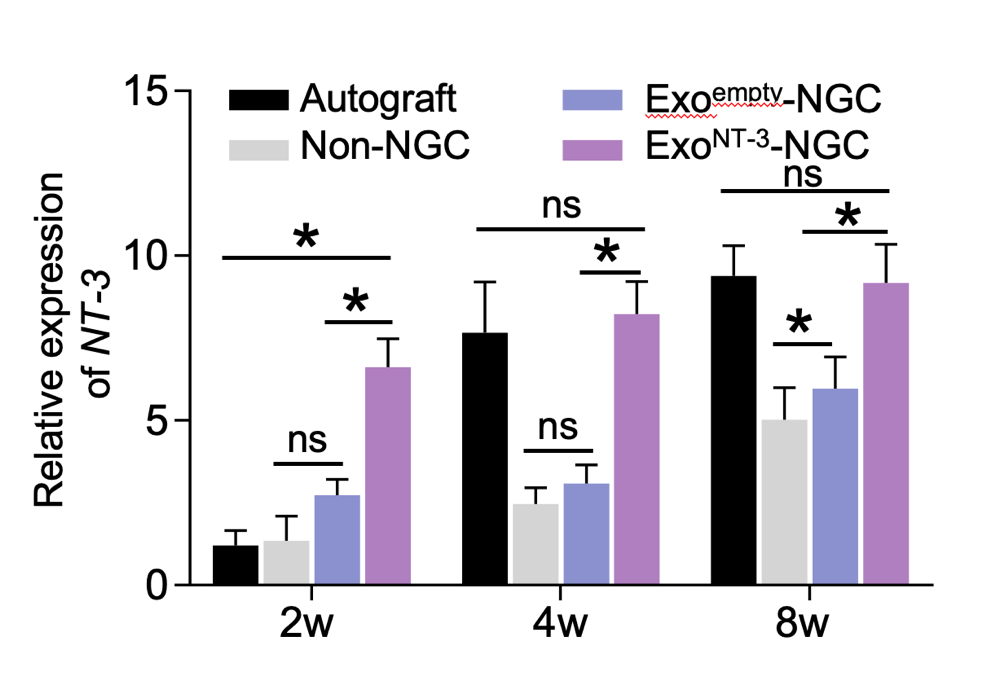


Figure S1. qPCR analysis of NT-3 mRNA expression level in the proximal segment of the transected nerves after 2, 4 and 8 weeks postoperation. Data are presented as mean ± SEM, n = 6 rats per group. **P* < 0.05 by one-way ANOVA with post hoc Bonferroni correction.
